# Genetic Modifier Screens Reveal New Components that Interact with the *Drosophila* Dystroglycan-Dystrophin Complex

**DOI:** 10.1371/journal.pone.0002418

**Published:** 2008-06-11

**Authors:** Mariya M. Kucherenko, Mario Pantoja, Andriy S. Yatsenko, Halyna R. Shcherbata, Karin A. Fischer, Dariya V. Maksymiv, Yaroslava I. Chernyk, Hannele Ruohola-Baker

**Affiliations:** 1 Department of Biochemistry, Institute for Stem Cell and Regenerative Medicine, University of Washington, Seattle, Washington, United States of America; 2 Department of Genetics and Biotechnology, Ivan Franko National University of Lviv, Lviv, Ukraine; University of Arkansas, United States of America

## Abstract

The Dystroglycan-Dystrophin (Dg-Dys) complex has a capacity to transmit information from the extracellular matrix to the cytoskeleton inside the cell. It is proposed that this interaction is under tight regulation; however the signaling/regulatory components of Dg-Dys complex remain elusive. Understanding the regulation of the complex is critical since defects in this complex cause muscular dystrophy in humans. To reveal new regulators of the Dg-Dys complex, we used a model organism *Drosophila melanogaster* and performed genetic interaction screens to identify modifiers of Dg and Dys mutants in *Drosophila* wing veins. These mutant screens revealed that the Dg-Dys complex interacts with genes involved in muscle function and components of Notch, TGF-β and EGFR signaling pathways. In addition, components of pathways that are required for cellular and/or axonal migration through cytoskeletal regulation, such as Semaphorin-Plexin, Frazzled-Netrin and Slit-Robo pathways show interactions with Dys and/or Dg. These data suggest that the Dg-Dys complex and the other pathways regulating extracellular information transfer to the cytoskeletal dynamics are more intercalated than previously thought.

## Introduction

Muscular dystrophies are a group of inherited neuromuscular disorders that share the same basic phenotype of progressive loss of muscle integrity. Many muscular dystrophies are caused by defects in a specialized cell adhesion complex called the Dystrophin Glycoprotein Complex (DGC). It has become evident that this complex plays a central role in muscle integrity and forms a mechanical link from the actin cytoskeleton to the extracellular matrix (ECM). The core DGC is composed of a transmembrane component, Dystroglycan (Dg), which associates with the ECM protein, Laminin and the cytoplasmic protein Dystrophin which binds Actin (reviewed in [1], [2]).

Many lines of evidence confirm that maintaining the structural link from the extracellular matrix to the actin cytoskeleton is crucial in preventing many forms of muscular dystrophy. Mutations that disrupt any component of this structural link results in a variety of muscular dystrophies like Duchenne's, Becker's, Muscle-eye-brain disease, Walker-Warburg syndrome, congenital muscular dystrophies 1C and 1D as well as limb girdle muscular dystrophy 2I. These diseases share the common symptoms of skeletal muscle degeneration, cardiomyopathy, as well as a reduced life span for afflicted individuals [3].

Additionally, alterations which reduce the affinity of components of the DGC lead to congenital muscular dystrophies like Fukuyama's which, aside from muscular defects, also are associated with aberrant neuronal migrations that lead to mental retardation, epilepsy, as well as abnormal eye development. The use of animal model systems has led to the clarification of the roles of specific gene products in maintaining muscle integrity and function (reviewed in [4]), however, the regulation of this complex is largely unknown.

**Figure 1:**
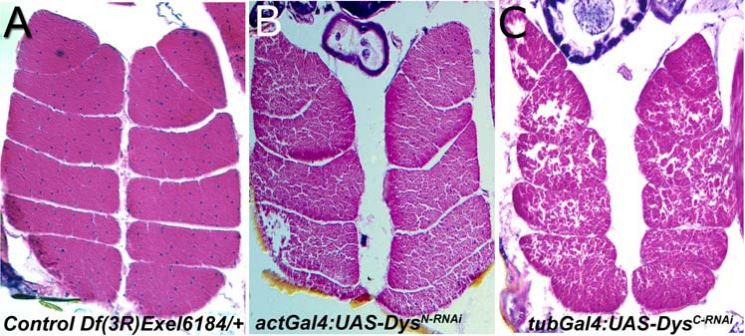
Muscle Phenotypes of *Dys* Mutants. Transverse histological sections of indirect flight muscles showing age dependent muscle degeneration. Control muscle section from *Df(3R)Exel6184/+* flies at 18 days of age do not show any type of abnormalities (A). While lower penetrance and milder muscle degeneration phenotype is observed from RNAi knockdown of long forms of *dystrophin* (*act-Gal4:UAS-Dys^N-RNAi^/+* flies at 18 days of age (B), *KX43/Df(3R)Exel6184* and *Dys8-2/KX43*), a strong muscle degeneration phenotype is seen when all forms of *dystrophin* are reduced (C; *tub-Gal4:UAS-Dys^C-RNAi^/+* flies at 12 days of age).

Initial characterization of the DGC in *Drosophila* has determined that components studied so far possess similar roles in muscle integrity and neuronal migration in flies as in humans (Figure 1, [5], [6], [7], [8]). These abnormalities include age dependent muscle degeneration, reduced mobility, defects in eye development as manifested by altered photoreceptor axon pathfinding, and a shorter life span. Additionally, mutations in *Dys* and *Dg* affect cell polarity in the *Drosophila* germ line as both the follicular cell epithelium and the oocyte are disrupted [6], [9], [10]. Recently, a reduced lifespan in *Drosophila*, as well as heart and muscle abnormalities, have been reported in mutants of another component of the DGC, *δ-*sarcoglycan [5]. In addition, heart and eye phenotypes have been observed in *Drosophila Dys* and *Dg* mutants [11], [12].

The similar defects in both flies and humans make *Drosophila* an attractive model for further studies on clarifying the role of the DGC. Such studies may reveal novel components that may likely have counterparts in humans. Additionally, since very little is known about how the DGC is regulated insights may be gained on this heretofore unknown process. More recently, transmembrane signaling has been implicated in the function of the DGC. The C-terminus of Dystroglycan, in addition to having, EF and WW domain binding sites, also possesses SH2 and SH3 domain binding sites. These known protein-protein interaction motifs support the idea that Dystroglycan is a signaling receptor in addition to its known role as a conduit between the ECM and the cytoskeleton. Changes in MAPK kinase and GTPase signaling have also been observed when the DGC is perturbed [2], [13]. Recent work has shown that specific sets of domains are critical in the function of *Drosophila* Dystroglycan [8].

**Figure 2:**
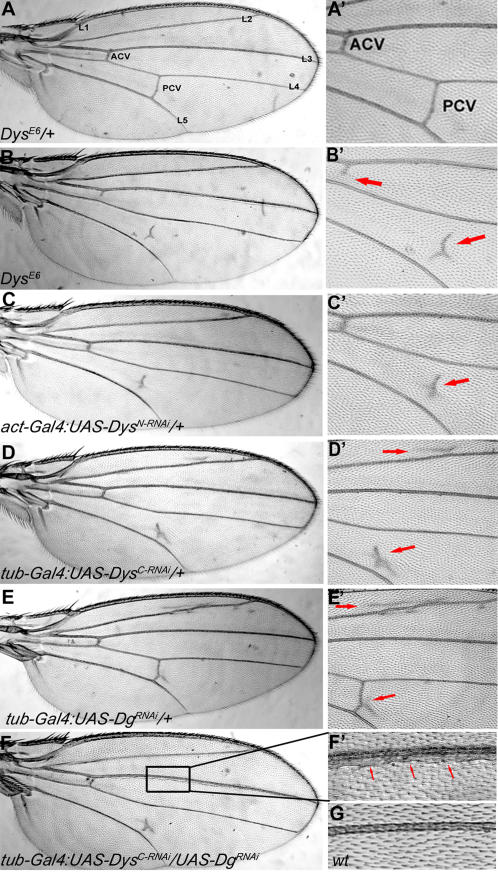
*Dys* and *Dg* are Required for Proper Wing Vein Formation and Interact in the Process. In a wild type like wing (A, genotype *Dys^E6^/+*) there are 5 longitudinal veins (L1–L5). The anterior cross vein (ACV) forms between L3 and L4 and the posterior cross vein (PCV) forms between L4 and L5. (A') Higher magnification of the region of the wing that shows both cross veins, ACV and PCV. *Dys^E6^/Dys^E6^* homozygotes show defects in cross vein formation (B). The PCV is detached from L4 and L5 and at a lower frequency, the ACV fails to form a connection to L4. (B') Higher magnification of the image in B where arrows indicate the altered ACV and PCV. Arrows indicate altered cross veins. The RNAi mutant that knocks down long forms of *dystrophin* (*act-Gal4:UAS-Dys^N-RNAi^/+*) also shows a PCV mutant phenotype where the cross vein fails to attach to L4 and L5 (C). (C') Higher magnification of the image in (C) where the arrow indicates the altered PCV. (D) Shows the wing vein phenotype of the RNAi *dystrophin* mutant (*tub-Gal4:UAS-Dys^C-RNAi^/+*) that reduces the protein levels of all isoforms. Here the PCV is drastically affected and there is extra vein material above L2. (D') Higher magnification of the image in (D) where the arrows indicate the alterations. The upper arrow shows extra wing vein material above L2. The lower arrow indicates an altered PCV. The RNAi *Dystroglycan* mutant (*tub-Gal4:UAS-Dg^RNAi^/+*) also shows a wing vein phenotype (E). In this case we see a branch off the PCV as well as extra material above L2. (E') Higher magnification of (E) where the upper arrow shows extra wing vein material above L2 and the lower arrow shows branching from the PCV. Finally, *Dys* and *Dg* interact in the *Drosophila* wing as the double mutant (*tub-Gal4:UAS-Dys^C-RNAi^/UAS-Dg^RNAi^*) shows a novel phenotype (F, F'). The box indicates a thickened L3 vein. (F') Higher magnification of the box is shown in (F). Arrows indicate extra L3 longitudinal vein material. (G) Wild type L3 vein from the same region as shown in (F').

In the present work, we have used the genetic tractability of *Drosophila* to search for novel components of the DGC, as well as components that may be involved in its signaling and regulation. Such a search is straightforward because in addition to the muscle degeneration and photoreceptor axon pathfinding defects, mutations in *dystrophin* and *Dystroglycan* cause a visible phenotype manifested as alterations in the fly wing, particularly the posterior crossvein (Figure 2, [14]). Since this is an easily score-able, highly penetrant phenotype we undertook a dominant modifier screen approach and looked for flies that showed either a suppression of the crossvein phenotype or a noticeably altered crossvein. Importantly, crossvein development has been previously shown to require EGFR, TGF-β and Notch pathway activities and is therefore a sensitive place to observe potential interactions of the DGC with these signaling pathways [15], [16], [17]. In addition, hemocyte migration is shown to correlate with the crossvein development [14]. Therefore genes involved in correct migration processes might also be obtained by this approach. We screened P-element lethal as well as deficiency collections for interactors in addition to performing a classical ethylmethanesulfonate (EMS) screen for dominant modifiers.

Here we report that in using these screening strategies we have found modifiers that belong to six different functional groups. We have found genes involved in muscle development, neuronal/cell migration and motor function as well as cytoskeletal components and components of the TGF-β, EGFR and Notch pathways. A common theme among many of these interactors and Dg-Dys complex is their involvement in the cytoskeletal rearrangements controlled by extracellular cues.

## Results

To identify genes that interact with *Dys* and *Dg*, we used the chromosomal lesion hypomorph *Dys^E6^* as well as the RNAi knockdown mutants *Dys^N-RNAi^, Dys^C-RNAi^* and *Dg^RNAi^*. These mutants exhibit age dependent muscle degeneration of indirect flight muscles (IFM, Figure 1B and 1C) as shown before for other *Dys* alleles [6].

In addition to the muscle phenotype, a visible wing vein phenotype was observed in these and previously analyzed alleles. In particular, the posterior crossvein (PCV) is defective in both *Dys* and *Dg* mutants, a disrupted anterior crossvein (ACV) and a partial duplication of L2, a longitudinal wing vein, can also be observed at a lower frequency (Figure 2). These phenotypes are present in the RNAi knockdown mutants, *Dys^N-RNAi^, Dys^C-RNAi^* and *Dg^RNAi^* (Figure 2C, 2D and 2E, respectively) as well as the chromosomal lesion hypomorphs *Dys^E6^* (Figure 2B), *DysDf, Dg^O43^, Dg^O55^* and *Dg^O86^* [14]. *Dys* and *Dg* are expressed in third instar larval wing imaginal discs (Figure S1) and interact in the wing vein since the double mutant shows a new phenotype – a duplication of L3 longitudinal wing vein (Figure 2F and 2F').

We used the visual wing vein phenotypes in P-element lethal, EMS induced and deficiency screens to find *dystrophin* and *Dystroglycan* interactors in *Drosophila* that either increase or decrease the wing vein phenotype. Additional studies reveal that many of the interactors identified in the screens are required for proper function in other tissues that require Dys and Dg, such as muscle, brain and ovary.

### Modifier Screens

To identify *Dys* and *Dg* interactors, we screened three different kinds of mutants; a collection of 800 FRT-P-element lethal lines, a deficiency collection of 216 lines and 43,000 EMS induced mutants. From these analyses, we identified 37 interacting genes that can be clustered into six different functional groups (Table 1).

**Table 1:** Modifiers of Dg-Dys Complex

Functional groups	Gene name	Allele(s)	Function	*act-Gal4: UAS- Dys^N-RNAi^/+*	*tub-Gal4: UAS- Dys^C-RNAi^/+*	*Dys^E6^/+ ^1^*	*Dg inte-ractors^2^*
**I. Muscle, motor and cyto-skeleton function**	***Dys***	*Dys[EMS-ModE10]*	Dys-Dg complex	-	-	En (S)	+
	***Cam***	*Cam[k04213]*	Calmodulin, muscle contraction	Su (W)	-	-	
	***mbl***	*l(2)k04222b* *	muscle development; splicing; RNA binding	Su (S)	Su+ (S)	-	
		*mbl[E27]* **		Su (S)	Su+ (S)	-	+
	***nAcRα-30D***	*l(2)k14204* *	acetylcholine receptor; muscle contraction	Su+ (W)	Su+ (W)	-	
		*nAcRα-30D[EY13897]* **		Su+ (W)	Su+ (W)	Mod (W)	+
	***Lis-1***	*Lis-1[k13209]*	Dynein binding, WD repeats	-	Su+ (M)	-	-
	***Khc***	*Khc[k13219]*	Kinesin, microtubule motor	-	Su+ (M)	-	-
	***Dmn***	*Dmn[k16109]*	Subunit in Dynactin complex	-	Su+ (M)	-	-
	***Fhos***	*l(3)j5B6*	Actin organizing protein	Su (M)	Su+ (W)	-	
**II. Neuro-nal migration or planar cell polarity genes**	***sema-2a***	*sema-2a[k13416]*	ligand of PlexinB, axon guidance	Su (W)	Su+ (W)	Mod (M)	+
	***sema-1a***	*sema-1a[k13702]*	axon guidance	En (M)	En (M)	-	+
	***fra***	*l(2)k03003* *	receptor of Netrin	Su+ (M)	Su+ (M)	Mod (W)	
		*fra*[*4*]**		Su (W)		Mod (W)	
	***sli***	*l(2)k02205* *	ligand (interects with Robo and Sdc)	Su (W)	Su+ (W)	Mod (W)	
		*sli*[*2*]**		Su (W)	Su+ (W)	-	-
	***robo***	*robo*[*2*]**	receptor	Su (W)	Su+ (W)	-	+
	***robo2***	*lea*[*2*]**	receptor		Su+ (W)	-	+
	***Sdc***	*l(2)k10317* *	Heparan sulphate proteoglycan (interacts with Sli and Robo)	Su+ (S)	Su+ (M)	-	
		*Sdc[10608]* **		Su+ (M)	Su+ (W)	-	+
	***stan***	*stan[129]* **	Receptor, Flamingo	Su+ (M)	Su+ (W)	-	+
	***wg***	*wg[spd-1]* **	ligand	Su+ (S)	Su+ (S)	Mod (M)	+
	***grh***	*grh[s2140]*	transcription factor	Su+ (S)	Su+ (S)	Mod (W)	+
**III. Notch signaling**	***Dl***	*Dl[EMS-Mod130]*	ligand of Notch	Su+ (S)	Su+ (M)	Mod (S)	+
		*Dl[EMS-Mod140]*		Su+ (S)	Su+ (M)	Mod (S)	+
**IV. TGF-β signaling**	***Dad***	*Dad[j1E4]*	negative regulator	Su+ (S)	Su+ (S)	Mod (M)	+
	***dpp***	*dpp[KG08191]*	TGF-β homolog	Su+ (M)	Su+ (W)	-	+
	***tkv***	*tkv[k16713]*	type I receptor	-	-	Mod (M)	
	***msk***	*msk[EMS-Mod90]*	importin	En (S)	En (S)	En (S)	+
		*msk*[*5*]**		Su (W)	Su+ (M)		+
**V. EGFR signaling**	***kek1***	*kek1[k07322]*	repressor of EGFR signaling	Su+ (S)	Su+ (M)	-	+
	***argos***	*l(3)j10E8* *	repressor of EGFR signaling; ligand	Su+ (W)	-	-	
		*argos[Delta7]* **		Su+ (S)	-	-	+
**VI. Other**	***Nrk***	*Nrk[k14301]*	receptor tyrosine kinase	Su (W)	Su+ (M)	-	-
	***HIPK***	*CG17090 [BG00855]*	serine/threonine kinase, death	Su+ (S)	Su+ (S)	Mod (S)	-
	***kis***	*kis [k13416]*	ATP helicase activity; chromatin binding	Su+ (S)	Su+ (S)	Mod (W)	+
	***gcm***	*gcm[KG01117]*	transcription factor activity	En (S)	En (M)	-	+
	***CG4496***	*CG4496[KG10365]*	zinc ion binding, nucleic acid binding	Su (S)	Su+ (S)		
	***wun***	*wun[k10201]*	lipid phosphate phosphatases	-	Su+ (W)	-	+
		*l(2)k11120a* *		Su+ (W)	-	-	
	***POSH***	*POSH [15815]*	SH3 adaptor protein, JNK signaling	Su (W)	Su+ (M)	-	+
	***vimar***	*vimar[k16722]*	Ral GTPase binding	Su (W)	Su+ (M)	-	+
	***del***	*del[KG10262]*	oogenesis	-	Su+ (S)		+
	***SP1070***	*Poly-EGF [EMS-Mod29]*	Notch binding (predicted)	Su+ (M)	Su+ (M)	-	
	***CG7845***	*CG7845[EMS-Mod4]*	WD40 domain protein	Su+ (M)	Su+ (M)	-	
	***l(3)L4092***	*l(3)L4092*	Zn-finger protein	Su+ (S)	Su+ (S)	Mod (S)	

* , P-element insertion used in screen that may affect gene;

** , gene allele from Bloomington stock center; S, suppressor; Su+, suppressor of PCV with extra wing vein material; En, enhancer; Mod, modifier; (W), weak; (M), moderate; (S), strong; 1, phenotypic classes shown in Figure S3; 2, summary for the data in Figure 4; +, interact; -, does not interact.

#### P-element screen

**Figure 3:**
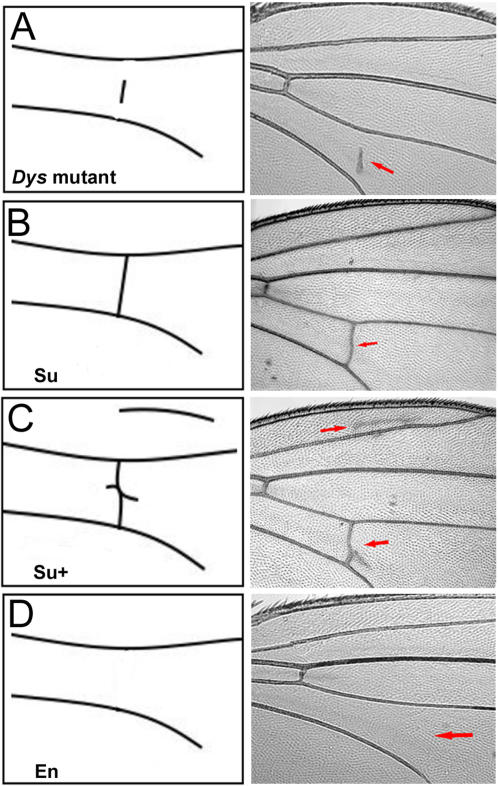
Wing Vein Phenotypes in *Dys* Modifier Classes. The posterior cross vein (PCV) was used in the screening process. The *Dys* mutant is depicted schematically in (A) with an actual fly wing (*act-Gal4:UAS-Dys^N-RNAi^/+*) shown to the right. Among the modifiers from the original mutant phenotype was the Su class or the completely suppressed class (B) where the PCV reverted to the wild type cross vein. Another class of interactors suppressed the detached PCV phenotype but also produced extra vein material, either as a branch or as an extra L2 vein (arrows, C). This group was classified as suppressor-plus (Su+). Finally, a group of modifiers showed a complete loss of the PCV (D, arrow) and this group was classified as enhanced (En).

**Figure 4:**
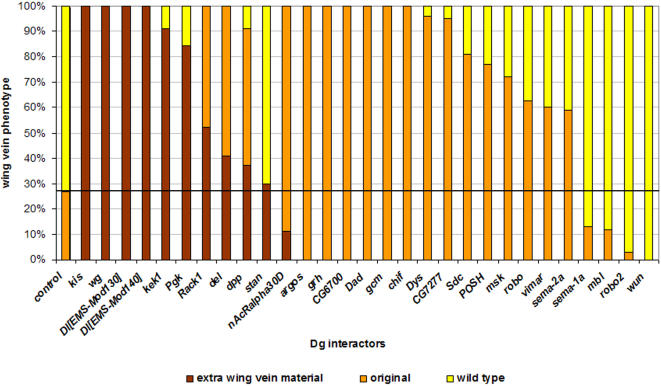
*Dg* Mutant Modifiers. The RNAi Dystroglycan mutant modifiers were scored either as an increase in penetrance of the Dg phenotype, or as an addition of extra vein material. These are represented in a bar graph. The ordinate indicates the percent penetrance of the wing vein phenotype. The abscissa indicates the genes that interact with *tub-Gal4:UAS-Dg^RNAi^/+* as heterozygotes. The unmodified *Dg* mutant phenotype is at the far left (control) and shows that nearly 30% of *Dg^RNAi^/+* flies show the Dg mutant phenotype (orange color) with the rest having wild type wing veins (yellow color). Modifiers that show an extra vein material are indicated in brown. Wing vein phenotypes are shown in Figure S4.

We screened FRT-P-element lethal lines from the Kyoto Stock Collection (Japan). The homozygous lethality of the lines allows us to infer that the transposon is inserted in or near an essential gene. We set up crosses to search for modifiers of the RNAi mutants *Dys^C-RNAi^* , *Dys^N-RNAi^* and *Dg^RNAi^* as well as *Dys^E6^*, a hypomorph that removes DLP2, a specific long isoform of *dystrophin* [7]. Modifiers were divided into phenotypic classes of enhancers, suppressors and suppressors with extra wing vein material (En, Su or Su+; Figure 3). From the P-element screen we obtained 33 modifiers of the *Dys* phenotypes (Table 1), 25 of which also showed interaction with Dg (Figure 4). Since the identity of these genes is known, we were able to organize these modifiers into 6 different functional categories (Table 1).

We were excited to find genes involved in both muscle and motor/cytoskeletal function as these are processes in which *Dys* and *Dg* are known to act (Table 1, functional group I; Figure 4). Of the muscle category genes, *muscleblind* (*mbl*) was the strongest suppressor in the wing vein. Weak suppressors of this group included *Calmodulin* (*Cam*) and the *nicotinic acetylcholine receptor α 30D* (*nAcRα-30D*) (Table 1, functional group I), though *nAcRα-30D* strongly interacted with *Dg* (Figure 4). Cytoskeletal and motor function genes (*Lissencephaly-1* (*Lis-1*), *Kinesin heavy chain* (*Khc*), *Dynamitin* (*Dmn*) and *Fhos* showed moderate to weak suppression with *dystrophin* mutants (Table 1, functional group I).

Another intriguing functional group that interacts with Dg-Dys complex includes genes known to be involved in neuronal migration (Table 1, functional group II). This group is composed of the genes which belong to Semaphorin-Plexin (*Sema-1a*, *Sema-2a*), Slit-Robo (*slit* (*sli*), *roundabout* (*robo*), *leak* (*lea*) – a robo2 homolog, *Syndecan* (*Sdc*)) and Netrin-Frazzlez (*frazzled* (*fra*)) pathways. In the wing vein most of these genes were scored as moderate to weak suppressors of the *Dys* RNAi mutants. Importantly, *Sema-2a* and *fra* modify *Dys^E6^* (Table 1, functional group II; Figure S3). *Sema-1a* was found to moderately enhance the *Dys* wing vein phenotype. Using our *Dg^RNAi^* mutant we found that *robo* and *Sema-2a* enhanced the *Dg* phenotype and *Sema-1a* and *lea* (robo2) suppressed the *Dg* phenotype (Table 1; Figure 4 and Figure S4). We have previously shown that *Dys* and *Dg* function in the *Drosophila* Photoreceptor axon pathfinding [6] in processes similar to Semaphorin-Plexin, Slit-Robo and Netrin-Frazzled pathways.

Functional groups IV and V (Table 1) contain interactors that belong to the TGF-β signaling pathway: *Daughters against dpp* (*Dad*), *decapentaplegic* (*dpp*), *thickveins* (*tkv*) and EGFR signaling pathway: *kekkon-1* (*kek1*) and *argos*. *Dad*, *dpp* and *kek1* were strong modifiers of the *Dg^RNAi^* mutant (Table 1; Figure 4 and S4) and *Dad* and *tkv* of *Dys^E6^* allele as well (Table 1, functional group IV; Figure S3). It has been known for some time that TGF-β, Notch and EGFR pathways are necessary for proper wing vein development ([18]; reviewed in [19], [20]). In fact, many novel factors of these pathways have been identified through the analysis of wing vein mutants. The longitudinal wing veins that begin to form during late larval stages of development require EFGR and TGF-β signaling pathways for proper fating of cells in the region [15], [21]. Crossveins appear in late pupal stages of development and require TGF-β signaling for formation and EGFR and Notch signaling for final patterning [17]. The posterior crossvein is particularly sensitive to different levels of TGF-β signaling and forms only after the proper formation of the longitudinal veins.

The last functional group (Table 1, functional group VI) contains a group of 12 genes with disparate functions. One enhancer, *glial cells missing* (*gcm*), was identified. Three strong suppressors of this group also interacted with *Dys^E6^*, the *Drosophila* homolog of the homeodomain-interacting protein kinase (HIPK), *kismet* (*kis*) and *l(3)L4092,* which contains a zinc-finger motiff. *kis*, *deadlock* (*del*), *gcm*, *POSH* and *wunen* (*wun*) also strongly modified *Dg^RNAi^* (Figure 4).

#### Deficiency screen

**Figure 5:**
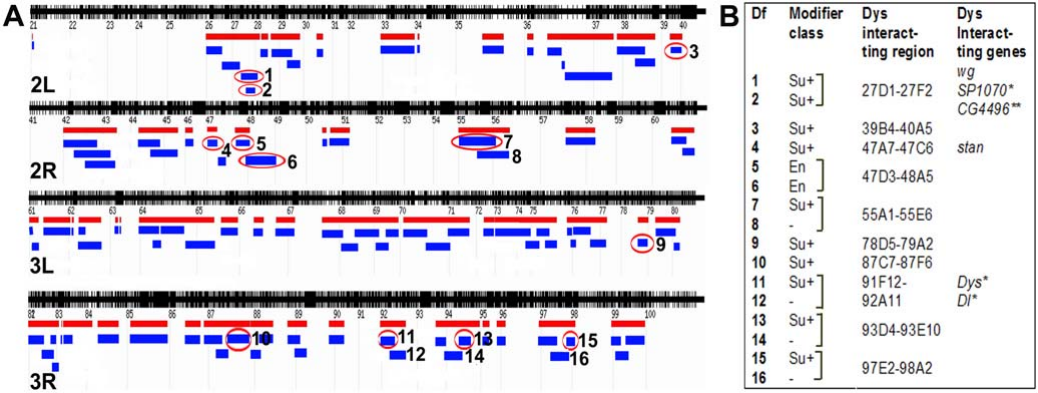
Deficiency Screen. (A) Cytological map of 2nd and 3rd chromosomes. Red bars represent cytological regions that were screened for interaction with *Dys* in wing vein. Blue bars represent deficiencies tested in the screen. Deficiences that showed interactions are circled in red. Numbers next to the blue bars indicate deficiencies used to narrow down the *Dys* interacting region. (B) Numbers in column 1 describe the deficiencies used to narrow down region and respond to the following deficiencies: (1) w^*^; Df(2L)spd^j2^, wg^spd-j2^, (2) *w^1118^; Df(2L)ade3*, (3) *Df(2L)ED1473*, (4) *Df(2R)ED2098*, (5) *Df(2R)en-B, b^1^ pr^1^*, (6) *Df(2R)en-A*, (7) *Df(2R)PC4*, (8) y^1^w^*^/Dp(1;Y)y^+^; Df(2R)P34, (9) *Df(3L)ED4978*, (10) *Df(3R)ED5612*, (11) *Df(3R)ED5942*, (12) *Df(3R)ED6025*, (13) *Df(3R)ED6069*, (14) *Df(3R)ED6076*, (15) *Df(3R)ED6265*, (16) *Df(3R)Tl-P, e^1^ ca^1^*. Class of wing vein phenotype modifiers is listed in second column. Cytology of 10 *Dys* interacting regions found in the screen is shown in column 3. Column 4 indicates Dys interacting genes found in deficiency, EMS (*) and P-element (**) screens.

In addition to the P-element lethal collection, we also screened for the *Dys* interactors using a collection of deficiency lines, which covers about 30% of the 1st, 40% of the 2nd and 80% of the 3rd chromosome and found 10 regions on the 2nd and the 3rd chromosomes that interacted with *Dys^N-RNAi^* in the wing vein (Figure 5A). Nine of these deficiencies suppressed the *Dys* PCV phenotype with formation of extra wing vein material and belonged to the Su+ class (Figure 3C and 5B) and one to the En class (Figure 3D and 5B). The *wg* and *stan* (*Flamingo, Fla*) genes, which belong to the planar cell polarity pathway (Table 1), were identified as *Dys^N-RNAi^* interactors in [27D1-27F2] and [47A7-47C6] cytological regions respectively (Figure 5B). Both of these genes modify *Dg^RNAi^* phenotype in wing vein as well (Figure 4). A third component of the planar cell polarity pathway, *grainy head* (*grh*), was identified from the P-element screen. Furthermore, *dystrophin* and *Dl* genes located in region [91F12-92A11], as well as *poly-EGF* and *CG4496* from [27D1-27F2] region were also independently identified in the P-element and EMS screens (Figure 5B and Table 1).

#### EMS mutagenesis screen

We analyzed ∼37,000 chromosomes for modifiers of *Dys^N-RNAi^* and *Dys^N2-RNAi^* and over 6000 chromosomes for enhancers of *Dys^E6^* and isolated 27 modifiers (Table S1). Eighteen of these were localized to the 2nd chromosome and nine were localized to the 3rd chromosome. The genes defective in eight of these mutants were identified and shown to correspond to five genes (Table 1). The modifier screens using EMS produced similar phenotypes to those observed in the P-element screen (Figure 4) and additionally produced a “posterior crossveinless” class (14/27 modifiers; Figure S2). These modifiers may be alleles of the *crossveinless* genes on the second and third chromosomes. There are eight previously identified *crossveinless* loci, *cv, cv-2, cv-3, cv-b, cv-c, cv-d, cvl-5* and *cvl-6*. Two, *cv* and *cvl-6*, are on the X chromosome. One, *cv-3* is on the second chromosome and the remaining five are on the third chromosome. Modulators of the TGF-β pathway are encoded by two of the eight *crossveinless* loci, *cv* and *cv-2*. Of the remaining six one other, *cv-c* has been molecularly characterized and encodes a *Drosophila* Rho GTPase Activating Protein. The others may also encode effectors of TGF-β signaling. Nevertheless, when *cv* and *cv-2* are lost the resultant aberrant TGF-β signaling results in the loss of both crossveins. Of our crossveinless like modifiers, one, Mod90 (Table 1 and Table S1), was mapped to cytological location 66B, two map units distal to the *hairy* gene, and so does not appear to be an allele of any of the *crossveinless* genes on the third chromosome. An attractive candidate for this gene is *moleskin* (*msk*) which encodes *Drosophila* Importin-7, a protein involved in nuclear translocation that has been shown to regulate the TGF-β pathway by controlling Mad localization. We further confirmed that *moleskin* (*msk^5^*) interacts with *Dg* and *Dys* (Table 1).

The next largest class of modifiers (9/27 EMS induced modifiers; Table S1) belonged to the Suppressor+ (Su+) class with extra wing vein material (Figure 3) and showed a more global effect on the wing. Four of these modifier mutants, Mod59, Mod111, Mod130 and Mod140, had phenotypes in the absence of the *dystrophin* mutant. We mapped them to cytological location 92A and showed that they are tightly linked to the *Delta* gene (*Dl*, Table 1). Subsequent crosses to different *Dl* alleles yielded lethal phenotypes, suggesting that these mutants are alleles of *Dl*. Two of the modifiers Mod130 and Mod140 suppressed the posterior crossvein phenotype of *Dys^N-RNAi^*, i.e. formed a complete crossvein from longitudinal vein L4 to longitudinal vein L5, in addition to generating extra wing vein material. We also found modifiers that belonged to the Enhancer as well as the Suppressor classes (Text S1).

Two other members of the class of mutants with extra wing vein material (Table S1 and Figure S2B), Mod4 and Mod29 were mapped to specific locations on the 2nd chromosome. Mod4 was mapped by following its lethality phenotype in crosses with deficiency lines. It was lethal in a cross with the *Df(2R)nap9/Dp(2;2)BG, In(2LR)Gla* line and was not lethal in a cross with the *Df(2R)ST1, Adh[n5] pr*[*1*] cn[*]/CyO line. The available lethal mutants from cytological region 42A1-42B3 were crossed to Mod4 and using a P-element insertion line, *PBac{PB}CG7845c00845/CyO* we determined that the mutation was in gene *CG7845.* This gene codes for a WD40 containing protein whose function is not known (Table 1). The underlying common function of all WD-repeat proteins is coordinating multi-protein complex assemblies, where the repeating units serve as a rigid scaffold for protein interactions. Such a scaffolding protein may be utilized in the formation or stabilization of the DGC.

Following the wing vein phenotype seen in homozygous flies as well as its semi-lethality, Mod29 was mapped to cytological region 27D1-27D4. This region was independently identified to contain a *Dys* interactors through the Deficiency screen (Figure 5B). We further fine-mapped the region by showing that two smaller deficiency lines, *Df(2L)ade3/CyO,P{ftz/lacB}E3* and *Df(2L)Exel7029,P+PBac{XP5.WH5}Exel7029/CyO* phenocopy the semi-lethality/wing vein phenotypes seen with Mod29. Ultimately, Mod29 was determined to be a *poly-EGF* gene mutant. Other genes in the region were eliminated by complementation analysis. In addition to the wing vein phenotype, Mod29 also shows defects in muscles, in oogenesis and in the brain (photoreceptor axon termination; Figure S5). Additionally, these mutant animals have a very short lifespan. Since Poly-EGF is predicted to bind the Notch receptor further functional analysis will be very interesting.

As discussed above, the *Dys^E6^* mutant does not show a wing vein phenotype as a heterozygote (Figure 2A). But as a homozygote it displays a disrupted posterior crossvein similar to *Dys^N-RNAi^* (Figure 2B and 2C). We reasoned that this heterozygote background was “sensitized” and therefore excellent for identifying enhancers. We screened 6000 chromosomes and found four modifiers for *Dys^E6^*/+. Three of those modifiers showed some extra wing vein material in the posterior crossvein (Figure S2B). Two of the modifiers, on the third chromosome ModE21 and ModE26 showed a branched crossvein phenotype in the absence of *Dys^E6^* though this dominant phenotype was not completely penetrant. The third of this group, ModE11 on the second chromosome, showed no wing vein phenotype in the absence of *Dys^E6^*, however as a homozygotes ModE11 flies displayed a globally disrupted wing vein characterized by an abundance of extra vein material (data not shown). Modifier E10 was the only modifier that showed an enhanced phenotype (Figure S3A). *ModE10/Dys^E6^* flies phenocopied the *Dys^E6^* homozygous phenotype which is a reduction in wing vein material (Figure 2B). Moreover, *ModE10/Df(3R)Exel6184* flies also gave a similar wing vein phenotype to *Dys^E6^/Df(3R)Exel6184* flies strongly arguing that ModE10 is a *dystrophin* allele. Finding an additional allele of *Dys* confirms that the screening approach was successful and analysis of the EMS mutant will allow us to further characterize Dystrophin protein function. Furthermore, the newly identified *dystrophin* allele *ModE10* interacted strongly with the *Dg* RNAi mutant in the wing; instead of 27% penetrance, the phenotype was 96% penetrant in heterozygous *Dys[Mod-E10]* background (Figure 4). This further confirms that molecules acting closely with Dys and Dg can be found in this assay.

### *mbl* Interacts with *Dys* in Muscles

These modifier screens led to the identification of genes that belong to different functional groups. Importantly, genes with known muscle function were identified in this screen, including *dystrophin* itself. In addition, the screen also identified muscle genes previously shown to interact with the Dg-Dys complex, *Cam*, *mbl* and *nAcRα-30D* [22], [23], [24]. *muscleblind* encodes a RNA binding protein that has shown to function in splicing [25]. As part of a secondary screen in muscle tissue, we tested whether *mbl*, a strong interactor with *Dys* in wing vein, also interacts with *Dys* in the indirect flight muscles.

**Figure 6:**
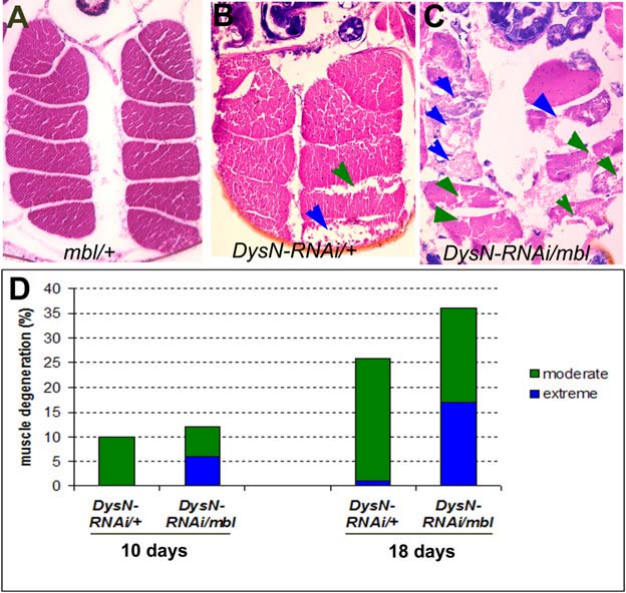
*mbl* Interacts with *Dys* in Muscle. (A–C) Transverse histological sections of indirect flight muscles of 18 days old flies. Reduction of *muscleblind* by one copy does not show obvious muscle defects (A). A stronger phenotype is observed in a *act-Gal4:UAS-Dys^N-RNAi^/+* mutant where loss of muscle integrity is noticeable throughout the tissue (B) and a significantly higher level of muscle degeneration is observed if the level of *muscleblind* is reduced in a *Dys* mutant background (*act-Gal4:UAS-Dys^N-RNAi^/mbl*, C). A green arrows in B and C indicate moderate muscle degeneration and blue arrows extreme muscle degeneration phenotype. The bar graph (D) quantifies the percentage of muscles that yielded the muscle phenotypes in 10 and 18 days old flies. Green bars indicate moderate muscle degeneration and blue arrows extreme muscle degeneration phenotype.

Animals that have lost one copy of *mbl* appear to have normal muscle structure (Figure 6A), while *act-Gal4:UAS-Dys^N-RNAi^* mutants show moderate muscle degeneration (10 days old: 10%, n = 68; 18 days: 25%, n = 159; Figure 6B and 6D). However, the extreme muscle degeneration phenotype was not observed in 10 days old and at a low frequency in 18 day old *Dys^N-RNAi^* mutants (Figure 6B and 6D). In contrast, when *mbl* was reduced by one copy in a *Dys^N-RNAi^* mutant background, increase in muscles defects was observed. Furthermore, around 50% of the phenotypes observed were classified “extreme” both in 10 and 18 day timepoints (Figure 6C and 6D). These data suggest that *mbl* and *Dys* interact in the muscles and reduction of *muscleblind* level enhances the abnormal muscle phenotype in *Dys^N-RNAi^* mutants. These results show that the screen successfully identified genes that interact with *dystrophin* to establish normal muscle function.

### Interactors Show Germline Phenotypes Similar to *Dys* and *Dg* Mutants

**Figure 7:**
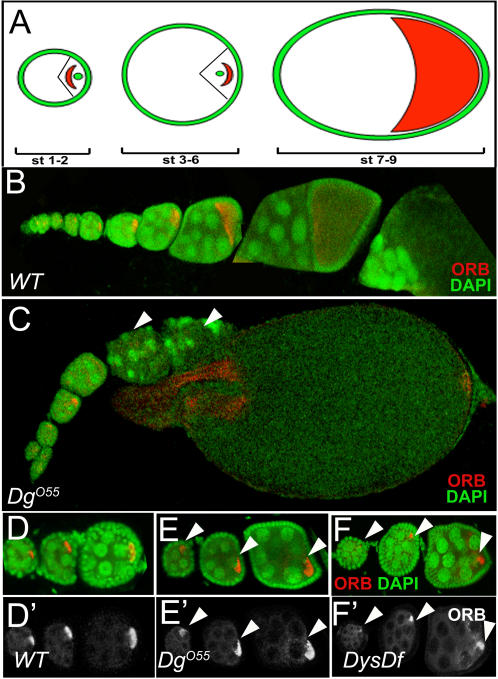
*Dys* and *Dg* Mutant Germline Phenotype. In the wild type ovariole, Orb localizes to the posterior of stage 3–6 oocytes (A, B). (C) In *Dg^O55^* mutants, however, there is mislocalization of Orb and much degeneration is observed. Arrowheads indicate mutant egg chambers. Additional analyses of *Dg^O86^*/*Dg^O55^*, *Dg^O43^*/*Dg^O55^* and *Dg^O86^*/*Dg^O43^* show similar phenotypes (not shown). Stage 3–6 wild type egg chambers show posteriorly localized Orb staining (D, arrowheads, red staining). (D') shows Orb staining alone. Stage 3–6 *Dg^O55^* mutant egg chambers show an abnormal lateral localization of Orb (E, arrowheads, red staining). (E') shows Orb staining alone. Stage 3–6 egg chambers from *DysDf* homozygotes where the Orb staining is defused. (E') more clearly shows the altered Orb staining.

The *Dys* and *Dg* genes are required in the germ line for the establishment of oocyte polarity [6], [8], [9]. The oogenesis defects of *Dys* and *Dg* serve as an excellent test to identify whether the new modifiers might interact with Dg-Dys complex in other tissues as well as in wing development. The ovariole contains a progression of egg chambers at different stages of development (Figure 7A). An early oocyte polarity marker, Orb, is localized to the anterior side in early stages and then migrates to the posterior side of oocyte by stage 3 of oogenesis. Between stages 3 and 6, Orb is clearly localized to the posterior of the oocyte, making it an excellent marker to analyze the polarity of the oocyte (Figure 7A–B). *Dg* loss-of-function germ line clones and homozygous point-mutants are arrested at early stages of oogenesis ([9]; Figure 7B) and show mislocalization of the Orb marker that is usually missing or diffused ([9]; Figure 7D). The oocyte polarity phenotype was also observed for viable *Dg* and *Dys* alleles (Figure 7B, 7D and 7E; 74%, n = 50 for *Dg^O86^/Dg^O55^*, 82%, n = 34 for *Dg^O43^/Dg^O55^* and 68%, n = 48 for *Dg^O86^/Dg^O43^*; the ovaries were dissected from 12 days old mutants).

**Figure 8:**
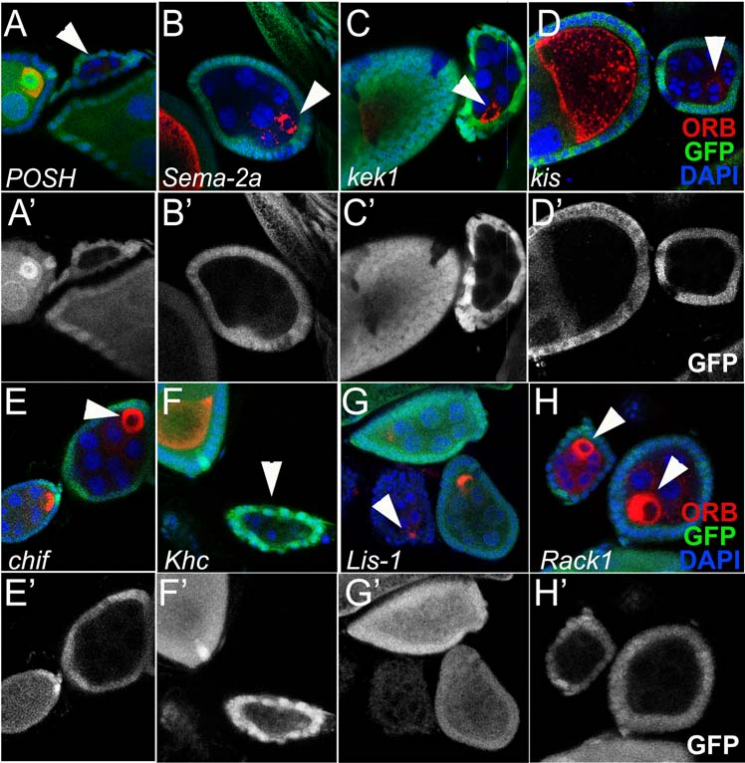
Interactors Show Similar Germ Line Phenotypes to *Dys* and *Dg.* * POSH* (*hsFlp, FRT42D*), loss of function clones show arrest at around stage 3–4 and appear to lack Orb staining completely (A). Loss of function clones of *Sema-2a* (*hsFlp, FRT42D Sema-2a[k13416]*) (B), *kek1* (*hsFlp, FRT40A kek1[k07322]*) (C), *kis* (*hsFlp, FRT40A kis[k13416]*) (D), *chif* (*hsFlp, FRT40A chif[BG02820]*) (E), *Khc* (*hsFlp, FRT42D Khc[k13219]*) (F), *Lis-1* (*hsFlp, FRT42D Lis-1[k13209]*) (G) and *Rack1* (*hsFlp, FRT40A Rack1[EY00128]*) (H) all show developmental arrest prior to stage 6. *Khc,* like *POSH* appears to lack any Orb staining (red) while the others have abnormal Orb staining ranging from punctate (*Sema-2a*) to completely surrounding the oocyte (*chif and Rack1*). GFP, green; Orb, red; DAPI, blue in (A–H). (A'–H') show GFP staining. Arrows indicate Orb staining in clones.

Interestingly, many of the identified modifiers are genes previously shown to be required in germ line development. For example a scaffolding protein, Rack1 that contains multiple WD-domains and interacts with atypical protein kinase C (aPKC) has recently been shown to function during *Drosophila* oogenesis [26]. We now analyzed potential Rack1 function in germ line for posterior Orb localization. Importantly, this analysis revealed that Rack1 is required in the early oocyte polarity: lack of Rack1 in early oocytes resulted in Orb mislocalization in the majority of the eggchambers (76%, n = 13, Figure 8H). Consistent with these findings aPKC is required for early oocyte development in *Drosophila* [27]. Similarly, another *Dys* and *Dg* interacting protein, Deadlock has previously shown to be required for Orb localization [28]. It is, therefore, likely that other identified *Dys* and *Dg* interactors are required in early oocyte polarity, possibly in a process closely related to Dg-Dys function in oogenesis.

Utilizing the FRT sites in the P-lethal lines we made germ line clones to determine how many of the selected modifiers had a role in the establishment of early oocyte polarity. We analyzed germ line clones of 14 loss-of-function mutants and found 8 genes that showed a similar phenotype to that seen in *Dg* loss-of-function clones. One of these genes, *POSH*, is an SH3 adapter protein. Loss-of-function clones of *POSH* (Figure 8A, white arrow) were arrested prior to stage 3–4 of oogenesis and appeared to lack Orb entirely. Orb mislocalization was also found in S*ema-2a*, *kek1, kis, Lis-1, chif* and *Khc*. *Sema-2a* loss-of-function clones showed clear developmental arrest prior to stage 4–6 with diffused Orb staining (Figure 8B). *kek1*, a negative regulator of the EGFR pathway, also showed developmental arrest in loss-of-function clones (Figure 8C). Orb in *kek1* clones surround the oocyte in an irregular pattern. *kis* loss-of-function germline clones have shown developmental arrest and diffused Orb staining (Figure 8D). Similarly, the majority of *Lis-1, chif* and *Khc* germline clones showed abnormal Orb localization (Figure 8E–8G). Lis-1 and Khc have previously shown to be involved in oocyte microtubular motor activities and to interact in this process [29], [30].

These data indicate that many of the genes that showed interactions with *Dys* and/or *Dg* in the wing vein assay, also showed early oocyte polarity phenotypes similar to *Dys* and *Dg* and potentially play a role in the establishment of early oocyte polarity.

## Discussion

In mammalian systems the DGC is composed of Dystrophin, Dystroglycan, the Sarcoglycan complex (α, β, γ, δ, ε, and ζ), Sarcospan, the Syntrophins (α and β), the Dystrobrevins (α and β) and Caveolin-3 [2]. Presently, the regulation of Dg-Dys complex and its involvement in signaling are poorly understood. In this work, we have addressed these unknowns by using dominant modifier screens to find genes that may shed light on both of these processes. Our screens have revealed groups of modifiers that are components of canonical signaling pathways (TGF-β, EGFR, Wnt and Notch) as well as components involved in cell/neuronal migration, cytoskeletal rearrangements and most importantly muscle development.

### New Components of the Dg-Dys Pathway?

The screens described in this paper revealed some expected interactors, *Dys*, *Cam* and *Khc*. Calmodulin, a calcium binding protein required for muscle and neuronal functions has previously been shown to interact with mammalian the Dg-Dys complex. However, whether reduction of *Cam* activities suppresses or enhances the muscular dystrophy phenotype is not totally clear. Targeted inhibition of *Cam* signaling exacerbates the dystrophic phenotype in mdx mouse muscle while genetic disruption of *Calcineurin* improves skeletal muscle pathology and cardiac disease in *ä-sarcoglycan* null mice [31], [32]. Since reduction of *Cam* showed suppression of the phenotypes caused by reduction of the long forms of *dystrophin* in the *Drosophila* wing, it will be interesting to analyze whether reduction of *Cam* will suppress the *Drosophila Dys* muscle phenotype as well. *Khc* involvement in Dg-Dys complex is also expected since work in mammalian system has shown that Khc can bind Dystrobrevin, a component of Dg-Dys complex [33]. It will be interesting to test in the future whether *Drosophila* Dystrobrevin can similarly bind Khc and what the functional significance of this interaction is in muscles and neurons. We have already shown that in oocyte development *Khc* is required for the same early as *Dys* and *Dg* (Figure 7). It is, therefore, interesting to test the potential requirement of *dystrobrevin* in this process and to further dissect the *Khc* function in this complex during early polarity formation.

An unexpected new interactor was identified in our screens, the homeodomain interacting protein kinase, HIPK. In mammalian systems HIPK is involved in the cell death pathway by phosphorylating p53 [34]. Recently *Drosophila* HIPK has shown to be involved in a communal form of cell death, sudden, coordinated death among a community of cells without final engulfment step [35]. It remains to be seen whether this HIPK communal death pathway will utilize p53 phosphorylation. However, it is tempting to speculate that the cell death observed in muscular dystrophies use the newly described HIPK dependent communal death pathway. HIPK is shown to interact with a WD40-protein in mammalian system [35]. Since three WD40 proteins were identified in our screens, it will be interesting to test whether any of these interact with *Drosophila* HIPK.

Another interactor that might shed light in the pathways utilized by the Dg-Dys complex is an SH3-domain adapter-protein, POSH. Structure-function analysis of Dg protein has revealed that a potential SH3-domain binding site in Dg C-terminus is essential for Dg function [8]. However, the critical SH3-domain protein in this complex is still at large. The present screen revealed that POSH can interact with the Dg-Dys complex in the wing vein assay. It will now be interesting to determine whether POSH is the missing critical SH3-domain protein that interacts with Dg-Dys complex in *Drosophila*.

### The Neuronal/Cell Migration Pathways Interact with the DGC

There are only a few examples of signaling pathways that have been shown to transmit information from outside the cell that results in cytoskeletal rearrangements inside the cell. Slit-Robo, Netrin-Frazzled and Semaphorin-Plexin pathways are examples of such activity. Dg-Dys complex appears also regulate the cytoskeleton based on extracellular information. Interestingly, the interaction screens described in this paper show that these aforementioned pathways are much more intricately connected than previously thought. The Robo and Netrin Receptor (DCC) pathways have previously been shown to interact [36], now we report that Dg-Dys complex interact with these pathways as well.

The interactions that we see in wing development involving the *Drosophila* DGC and the genes that affect neuronal guidance (*sli*, *robo*, *fra*, *sema-2a*, *sema-1a*, *Sdc*) might be explained by their possible role in hemocyte (insect blood cell) migration. Analysis done in *Drosophila* shows that known axon guidance genes (*sli, robo*) are also implicated in hemocyte migration during development of the central nervous system [37]. Similar findings have been reported in mammals, where blood vessel migration is linked to the same molecular processes as axon guidance [38]. Both *sli* and *robo* have been implicated in the vascularization system in vertebrates [39]. A recent study demonstrated that proper hemocyte localization is dependent upon *Dys* and *Dg* function in pupa wings. Mutations in these genes result in hemocyte migration defects during development of the posterior crossvein [14]. Hence, we speculate that the neuronal guidance genes that we have found may interact with the DGC in wing veins by having a role in the migration process.

Similar to *sli* and *robo* [40], the *Dys* and *Dg* mutants also affect photoreceptor axon pathfinding in *Drosophila* larvae [6]. It is therefore possible that this group of modifiers will interact with the DGC in axon pathfinding and other processes. Supportive of that notion is the fact that mammalian Syndecan-3 and Syndecan-4 are essential for skeletal muscle development and regeneration [41]. In addition *slit-Dg* interaction has previously been observed in cardiac cell alignment [42]. Sequence analysis of slit reveals that it possesses a laminin G-like domain at its C-terminus. Dystroglycan's extracellular domain has laminin G domain binding sites and has been shown to bind 2 of the five laminin G domains in laminin. It is therefore possible that slit, through its laminin G-like domain, binds to Dystroglycan and that Dystroglycan is a slit receptor. It will be informative to reveal the mechanisms and nature of these interactions.

### Oocyte Phenotypes

The establishment and formation of oocyte polarity during development is a complex multistep process (reviewed in [43]). In the anterior part of the germarium each stem cell undergoes asymmetric cell division to give rise to another stem cell and a cystoblast. The cystoblasts divide four times with incomplete cytokinesis to form a 16 cell cyst. The oocyte fate is determined when the cyst reaches the end of the germarium. At this point, BicD protein, Orb protein, the microtubule organizing center (MTOC) and the centrioles move from the anterior to the posterior of the oocyte (Figure 7A). These events mark the first sign of polarity in the oocyte. Subsequent Gurken signaling induces posterior follicle cells to signal back to the oocyte which repolarizes the microtubule cytoskeleton. This signal appears to require an intact extracellular matrix since *Laminin A* mutants do not undergo repolarization [44]. The outcome of the repolarization results in the disassembly of the MTOC at the posterior, nucleation of microtubules anteriorly and subsequent migration of the oocyte nucleus to an antero-lateral position.

Germ line clones that lack *Dg* show developmental arrest and mislocalization of the oocyte polarity marker Orb which is usually diffused or absent in the oocyte [9]. This phenotype might be due to *Dg* affecting the localization of the MTOC. But how exactly *Dg* is involved in this process is not clear. One possible explanation is that Dg is required for extracellular matrix (ECM) integrity since it is known to bind Laminin. Such a structural conduit may be necessary for proper signaling from the posterior follicle cells to the oocyte. This is supported by the fact that *Dg* loss-of-function mutants show defects in Actin accumulation. Another possibility is that *Dg* may be involved in mircrotubule organization. Since the regulation of actin- and microtubule-cytoskeleton are connected, these two models are not mutually exclusive.

Interestingly, in our screens we found several genes that showed phenotypes in oocyte development. One of these genes is *kek1*, a transmembrane protein of the *Drosophila* Kekkon family that has been reported to be a negative regulator of the EGFR receptor [45]. It has been previously shown that EGFR signaling regulates the expression pattern of Dystroglycan to establish anterior-posterior polarity of oocyte [10]. Further study is required to determine if *kek1*, as an EGFR regulator controls *Dg* expression in the germ line.

Another interesting gene found in our screens is *POSH* (Plenty of SH3 domains), a *Drosophila* homologue of human SH3MD2 protein. Interestingly POSH is a multidomain scaffold protein that can interact with Rho related GTPase - Rac1 and promotes the activation of the JNK pathway [46] POSH has also shown to regulate POSH-MLK-MKK-JNK complex [47]. A defect in this complex can affect brain function. Furthermore, POSH and JNK-mediated cell death pathway is thought to play an important role in Parkinson's disease [48]. With many SH3 domains, POSH has the potential to bind *Dg* that has a predicted SH3-domain binding site and has been shown to be necessary for the establishment of oocyte polarity [8].

In addition, we have found interactions with *Khc*, *Lis-1* and *Dmn*, three genes known to be part of the Dynein-Dynactin complex which in addition to Kinesin microtubule motor activity have been shown to be necessary for establishment of intracellular polarity within the Drosophila oocyte [49], [50]. In mid-oogenesis dynein, dynactin and kinesin are thought to act cooperatively in cargo transport [49], [51], [52]. Since these genes interact with *Dys* (Table 1) and show similar phenotypes in Orb localization (Figure 7), it will be interesting to dissect their potential functional interactions with *Dys* in early oocyte development. Furthermore, since mammalian Dystrobrevin physically interacts with Khc, it is plausible, that the Dynein-, Dynactin-, Kinesin-complex will utilize localization cues set-up by Dg-Dys Complex.

In addition to the interactions with microtubular motor-complexes, we also found interactions with a *Drosophila* Formin homologue, FHOS. Mammalian FHOS directly binds to F-actin and promotes actin fiber formation [53]. Recently *Drosophila* actin nucleators, Capu and Spire have shown to assemble a cytoplasmic actin mesh that maintains microtubular organization in the middle of oogenesis [54], [55], [56], [57], [58]. Therefore, it will be important to determine whether the actin nucleator, FHOS is also involved in actin nucleation that regulates microtubular activity in early oocyte development. Further study of these cytoskeletal genes will allow us to gain a more detailed understanding of how *Dg* and *Dys* function to ensure proper oocyte polarity during oogenesis.

Similar to microtubule and actin interplay in the regulation of oocyte polarity, the dynamic actin-microtubule interactions regulate growth cone steering at the growing axons [59]. It is therefore possible that similar mode of function for Dg-Dys interactions with these cytoskeletal modules is used in various cell types. Furthermore the axon pathfinding and oocyte polarity formation processes are similar in that they are controlled by extracellular information which is transmitted to the cell resulting in cytoskeletal rearrangement.

### Planar Cell Polarity Genes

At the basal side of follicle epithelium, actin filaments exhibit a planar cell polarity that is perpendicular to the long axis, the AP axis, of the egg chamber. In *Dg* follicle cell clones the basal actin array is disrupted non-cell-autonomously [9]. Integrins and the receptor tyrosine phosphatase *Lar* are also involved in basal actin orientation [60]. It is unclear whether *Dg* and the other genes involved in basal actin polarity act together with the canonical planar cell polarity pathway or function independently of this pathway. Interestingly, we now report strong interactions between the DGC and *grainy head* (*grh*) a transcription factor which is required for several different processes during the differentiation including the function of the *frizzled* dependent tissue polarity pathway, epidermal hair morphogenesis and wing vein specification [61]. In the absence of *grh* function the Fz, Dsh and Vang proteins fail to accumulate apically and the levels of Stan (or Flamingo) protein are dramatically decreased. The interactions seen with *stan* (*Fla*) and *wg* in wing veins supports the hypothesis that Dg might act together with the *frizzled*-dependent tissue polarity pathway in coordinating the polarity of cells in epithelial sheets.

### Conclusions

By screening for alterations of a dominant wing vein phenotype we have found modifiers of the DGC that are involved in cytoskeletal organization. Initial characterization of some of these genes revealed that they have phenotypes also in other tissues, in which the DGC is known to function. These tissue/cell types include the oocyte, the brain and the indirect flight muscles. This argues strongly that the identified interactors may be involved globally in DGC function. Further study is required to determine mechanistically how these modifiers work in the context of the Dg-Dys complex. However a common theme, already arising is that the identified interactors appear to regulate cytoskeletal rearrangement. Mechanistic understanding of how the new interactors might regulate Dg-Dys communication with cytoskeleton of muscle cells may serve as a basis for the development of novel therapeutic approaches that might improve the quality of life of individuals afflicted with muscular dystrophy.

## Materials and Methods

### Fly Strains and Genetics

The fly-strains used is in this study are: *Dys^E6^/TM3, UAS-Dys^N-RNAi^/CyO* (both kindly provided by L.Fradkin and described previously as *dys^DLP2 E6^* and *RNAi-dysNH_2_* [62]), *UAS-Dys^N2-RNAi^/TM3, UAS-Dys^C-RNAi^/TM3, UAS-Dg^RNAi^/TM3* (described previously as *UASdsDysN-term*, *UASdsDysC-term* and *UASdsDg*, respectively [6]), *DysDf, Dg^O43^/CyO, Dg^O55^/CyO, Dg^O86^/CyO* (kindly provided by R. Ray [14]), *KX43/TM6C, Dys8-2/TM3* [6], *act-Gal4/CyO, tub-Gal4/TM3, Ubi-GFP FRT42D,Dg^323^/CyO, hsFLP; FRT40A Ubi-GFP/CyO, hsFLP; FRT42D Ubi-GFP/CyO* and *hsFLP; FRT82B/TM3.* Alleles used in this study *w[1118]*, *msk*[*5*]*, nAcRα-30D[EY13897], fra*[*4*]*, mbl[E27], sli*[*2*]*, robo*[*2*]*, lea*[*2*]*, Sdc[10608], stan[129], wg[spd-1], argos[Delta7]* and *Df(3R)Exel6184,* the deficiency for *dystrophin* were obtained from Bloomington Stock Center.

The following *Dys* and *Dg* mutants were used to screen for modifiers of the wing vein phenotype: *Dys^E6^*, an hypomorphic allele of *dystrophin, act-Gal4:UAS-Dys^N-RNAi^/CyO, tub-Gal4:UAS-Dys^N2-RNAi^/TM3, tub-Gal4:UAS-Dys^C-RNAi^/TM3* which are three *Dys* RNAi mutants recombined onto chromosomes with actin and tubulin Gal4 drivers, respectively; and *tub-Gal4:UAS-Dg^RNAi^/TM3*, a *Dg* RNAi mutant recombined onto a chromosome with the tubulin Gal4 driver. *Dys^N-RNAi^* is a transgene on the second chromosome that encodes an inverted repeat that forms a double stranded RNA hairpin complementary to the corresponding N-terminus of the protein. It reduces the protein levels of all the known long isoforms. *Dys^N2-RNAi^* is a transgene on the third chromosome that encodes a different inverted repeat than *Dys^N-RNAi^* but still reduces the protein levels of the known long isoforms. *Dys^C-RNAi^* is a transgene on the third chromosome that encodes sequence complementary to the corresponding C-terminal region of *dystrophin* and reduces the protein levels of all isoforms. All *Dys* RNAi mutants exhibited 100% penetrance of the wing vein phenotype. The *Dg* RNAi mutant exhibited ∼30% penetrance of the wing vein phenotype.

#### P-element screen

The FRT P-element insertion lethal lines used in this study were obtained from the Kyoto Stock Center (Japan). From this collection, 800 lines were screened. Two-to-four day-old males carrying P-lethal insertions over balancers (or virgin females if the P-element was on the X chromosome) were mated to *Dys^E6^/TM3; act-Gal4:UAS-Dys^N-RNAi^/CyO; tub-Gal4:UAS-Dys^C-RNAi^/TM3* and *tub-Gal4:UAS-Dg^RNAi^/TM3* virgin females (or males). Non-balancer F_1_ progeny were screened for dominant modification of the wing vein phenotype. Modifiers were divided into phenotypic classes based on the alterations of their wing veins as compared to wing veins of *act-Gal4:UAS-Dys^N-RNAi^/(+ or CyO), tub-Gal4:UAS-Dys^C-RNAi^/(+ or TM3)* and *tub-Gal4:UAS-Dg^RNAi^/(+ or TM3)* sibling flies. Suppressors were identified when wing veins of F_1_ flies phenocopied the wing veins seen in wild type flies. Since *Dys^E6^*/+ had wild-type wing veins, non-balancer F_1_ progeny from the *Dys^E6^/TM3* and P-lethal insertion cross were analyzed for the presence or absence of a mutant posterior crossvein which is altered in *Dys^E6^* homozygotes. All the genes found in this screen were crossed to *w[1118]* to verify the absence of a dominant wing vein phenotype. None modifiers showed a dominant posterior wing vein phenotype. Since P-element insertion lethal lines were on chromosomes with FRT sites, we made mosaics and determined if selected modifiers possessed an oocyte polarity phenotype (see below).

#### EMS screen

For the EMS mutagenesis 2–4 day old *w^1118^* males were place in yeasted bottles overnight. Twenty males were placed in each vial and starved for 6–8 hours. The flies were then provided access to a solution of 22–25 mM EMS (ethyl methansulfonate; Sigma) in a 5% sucrose solution for 18 hrs. The flies were placed in new vials and allowed to recover for 1–3 hrs. Each vial of flies was transferred to yeasted bottles with 40 virgin females of the desired genotype per bottle. The females were allowed to lay eggs for two days then transferred to new bottles daily for two more days. Mutagenized *w^1118^* males were crossed to *act-Gal4:UAS-Dys^N-RNAi^/CyO* or *tub-Gal4:UAS-Dys^N2-RNAi^/TM3* females. Males of these crosses were scored for a modification of the wing vein phenotype, either a novel alteration or suppression. These were picked and backcrossed to the parental *Dystrophin* mutant females to determine whether the modification was on the second or third chromosomes. Once this was done these modifiers were balanced either with *CyO* or *TM6B* and maintained as a stock. The subset of modifiers that produced phenotypes in the absence of the *Dys* mutants were crossed to meiotic mapping stocks and subsequently mapped using those phenotypes. The remainders were crossed to meiotic mapping stocks with a *dystrophin* mutant in the background and mapped using the modification phenotypes. In addition to the two RNAi *dystrophin* mutants mentioned above, enhancers of the *Dys^E6^* mutant were also screened for.

Obtained modifiers were mapped meiotically using the lines from Bloomington Stock Center *al*[*1*]* dp[ov1] b*[*1*]* pr*[*1*]* c*[*1*]* px*[*1*]* sp*[*1*] and *ru*[*1*]* h*[*1*]* th*[*1*]* st*[*1*]* cu*[*1*]* sr*[*1*]* e[s] ca*[*1*] .

#### Deficiency screen

A collection of 216 deficiency lines were screened for modifiers of *act-Gal4:UAS-Dys^N-RNAi^/CyO* mutant. These lines were a mixed collection of flies from the Bloomington Stock Center, the Exelixis (Harvard) and Drosdel (Cambridge) collections. Additionally, these lines were used for mapping modifiers (Mod29 and Mod4) from the EMS screen. Deficiencies which show interaction in screen: w^*^; Df(2L)spd^j2^, wg^spd-j2^; *w^1118^; Df(2L)ade3*; *Df(2L)ED1473*; *Df(2R)ED2098*; *Df(2R)en-B, b^1^ pr^1^*; *Df(2R)en-A*; *Df(2R)PC4*; y^1^w^*^/Dp(1;Y)y^+^; Df(2R)P34; *Df(3L)ED4978*; *Df(3R)ED5612*; *Df(3R)ED5942*; *Df(3R)ED6025*; *Df(3R)ED6069*; *Df(3R)ED6076*; *Df(3R)ED6265*; *Df(3R)Tl-P, e^1^ ca^1^.*

### Loss of Function Mosaic Analysis

To obtain germline clones of the modifiers found in the FRT P-element lethal screen, they were crossed to corresponding chromosomal FRT stocks. Third instar larvae and early pupae from this cross were heat-shocked at 37°C for 2 hours. After eclosion, they were placed in vials with fresh yeast paste for 4–5 days before dissection.

### Dissections and Immunohistochemistry

*Drosophila* ovaries and wing imaginal discs were dissected rapidly in phosphate buffered saline (PBS) and fixed in 4% paraformaldehyde (PFA) for 10 minutes at room temperature. Adult's brains were dissected in PBS and fixed in PFA for 30 minutes. All antibody stainings were performed as described previously [6], except with the Dystrophin antibody with which a protocol provided by L. Fradkin was used [62]. Ovaries, brains and wing imaginal discs were mounted in 70% glycerol in PBS for analysis using a Leica TSC SPE confocal microscope. The following antibodies were used: rabbit anti-Dg (1∶3000; [9]), anti-Dys CO_2_H (1∶3000; [62]), mouse anti-Orb (1∶20; Developmental Studies Hybridoma Bank), mouse anti-24B10 (1∶20; Developmental Studies Hybridoma Bank), Alexa 488, 568, or 633 goat anti-mouse, Alexa 488, 568 goat anti rabbit. To mount adult wings, flies were pre-incubated in 80% ethanol and 20% glycerol solution, then dissected and mounted in 70% glycerol before analysis using a Leica light microscope.

### Muscle Analysis

Histological sections of muscle were prepared from wax-embedded material as described previously [6], stained with hematoxyline and eosin (H&E staining) and analyzed using light microscopy.

## Supporting Information

Text S1 (0.03 MB DOC)

Table S1 (0.14 MB DOC)

Figure S1 Dys and Dg Expression in Wild Type and Mutant Wing Discs. In wild-type larvae, Dys is expressed in all cells of the wing disc (A) and is strongly reduced in the wing disc of the DysC-RNAi mutant (B; tubGal4:UAS-DysC-RNAi/+). Dystroglycan localization in wild type imaginal discs is enriched at the basal surface of the epithelial cells. Dg expression is more intense in folds formed from the contact of basal surfaces and is less visible in the folds made from apical surfaces of wing disc cells (C). The DgRNAi mutant also shows a reduction of Dystroglycan protein in the wing disc (D; tub-Gal4:UAS-DgRNAi/+). (A′–D′) Enlarged images of framed areas on (A–D). (A"–B") Enlarged images of framed areas on (A–B) show Dystrophin single channel staining. (C"–D") Enlarged images of framed areas on (C–D) show Dystroglycan single channel staining.
(6.06 MB TIF)

Figure S2 Subclasses of Dys(RNAi) Modifiers. (A) Example of enhancers that lack the posterior cross vein (PCV) and belong to phenotypic class En. Arrow indicates where the PCV should be. (B) Wing representing Su+ class phenotype that shows the PCV attached to L4 and L5 and extra wing vein material can be seen below L5.
(0.94 MB TIF)

Figure S3 DysE6/+ Modifiers. DysE6/+ flies have normal posterior cross veins. (A) Represents an enhancer (ModE10) of this phenotype which phenocopies the wing veins from DysE6/DysE6 flies. (B) Shows an extra vein modification of the DysE6/+ posterior cross vein. Arrows indicate altered cross veins.
(1.94 MB TIF)

Figure S4 Dg(RNAi) Modifiers. (A) Wild type fly wing with normal posterior cross vein (PCV) as indicated by the arrow. (B) DgRNAi mutant PCV (arrow) with a branch. (C–D) represent modifiers that produce extra vein material (indicated by arrows. In one case (C) the branch is elongated with extra material also seen above L2 (upper arrow). (D) Shows extra material below L5 (lower arrow).
(1.77 MB TIF)

Figure S5 Phenotypes Observed in poly-EGF Mutants. (A) Fly wing from Mod29/Df flies. Arrows indicate extra vein material. (B) Transverse histological section of the indirect flight muscle in Mod29 homozygotes. (B') Higher magnification of the region indicated by the box which indicates the separation of the muscle into individual fibers. (C) Ovariole from a Mod29 homozygote. Anterior to the left. The germarium is at the anterior most tip of the ovariole with developing egg chambers progressing to the right (posteriorly). The egg chambers undergo developmental arrest in later stages. Actin-Green, Adducin-Red, DAPI-purple. (D) 24B10 antibody staining of the adult wild type brain. Arrows indicate photoreceptor axon termination sites where the R8 photoreceptor axon (left arrow) terminates before the R7 axon (right arrow). (E) Mod29/Df adult brain. Termination of the R8 and R7 axons are indicated by the red arrows. White arrows indicate non termination of two axons that protrude deeper into the brain. (F) Dg323/Dg323 clone in the adult brain. Red arrows indicate where the R8 and R7 axons should terminate. Upper white arrows indicate a general disruption of axon termination in the R8/R7 termination region. Lower right arrow indicates a non terminating axon that proceeds deeper into the brain.
(3.58 MB TIF)
