# Supplementary material for: Genetic Modifier Screens Reveal New Components that Interact with the Drosophila Dystroglycan-Dystrophin Complex
Source: PLoS One. 2008 Jun 11;3(6):e2418. doi: 10.1371/journal.pone.0002418 (PMC2398783; doi:10.1371/journal.pone.0002418)
Supplement: Text S1 — (0.03 MB DOC) [file pone.0002418.s001.doc]

**Text S1**

**EMS Screen**

There were EMS induced modifiers (3/27) that belonged to the enhancer class (En). These were identified due to a reduction in posterior crossvein material and resulted in a “dot” like phenotype (Figure S2B). These mutants (Mod55, Mod61 and Mod121) in the absence of the *Dystrophin* mutant exhibited crossvein phenotypes similar to *Dys* mutant phenotypes, though they were not completely penetrant. Therefore the observed modification phenotypes may be additive.

The EMS screen yielded one modifier, Su5 that completely suppressed the *Dys* mutant wing vein phenotype (Figure 3B). Crosses of Su5 with *Dys^N-RNAi^* and *Dys^N2-RNAi^* yielded complete suppression in 70-75% of the progeny. In crosses of Su5 with *Dys^C-RNAi^* complete suppression was less penetrant (~1%), but the wing veins of the other progeny were more “wild type” than *Dys^C-RNAi^* alone. It is unlikely that this suppression is due to mutations in the siRNA pathway since double heterozygotes with the *Dicer-2* mutant did not affect wing vein morphology.
