# Supplementary material for: Genetic Modifier Screens Reveal New Components that Interact with the Drosophila Dystroglycan-Dystrophin Complex
Source: PLoS One. 2008 Jun 11;3(6):e2418. doi: 10.1371/journal.pone.0002418 (PMC2398783; doi:10.1371/journal.pone.0002418)
Supplement: Table S1 — (0.14 MB DOC) [file pone.0002418.s002.doc]

**Table S1.** *Dys* EMS Modifiers

| **Phenotypic**  **group^a^** | **Modifiers^b^** | **Chromo-some** | **Arm** | **Cyto-logy** | **Gene** | **Homo-zygous**  **lethal^c^** | **Homo-**  **zygous**  **fertile^d^** | **Recessive**  **wing vein**  **phenotype^f^** | **Homozy-**  **gous**  **lifespan^g^** |
| --- | --- | --- | --- | --- | --- | --- | --- | --- | --- |
| ***Dys^N-RNAi^, Dys^N2-RNAi^*** | | | | | | | | | |
| Su | Su5 | 2nd | 2L |  |  | + | - | - |  |
| En | Mod3 | 2nd |  |  |  | - | + | - |  |
| Su+ | Mod4 | 2nd | 2R | 42A | *CG7845* | + | - | - |  |
| En | Mod5 | 2nd |  |  |  | - | + | - |  |
| En | Mod10 | 2nd |  |  |  | + | - | - |  |
| En | Mod12 | 2nd |  |  |  | - | + | - |  |
| En | Mod13 | 2nd |  |  |  | - | + | - |  |
| En | Mod17 | 2nd |  |  |  | - | + | - |  |
| En | Mod19 | 2nd |  |  |  | - | + | - |  |
| En | Mod20 | 2nd |  |  |  | - | - | - |  |
| Su+ | Mod29 | 2nd | 2L | 27D | *poly-EGF* | (+) | - | ++++ | 7 days |
| En | Mod30 | 2nd |  |  |  | - | + | - |  |
| En | Mod31 | 2nd |  |  |  | - | + | - |  |
| En | Mod35 | 3rd | 3L |  |  | + | - | - |  |
| En | Mod55 | 2nd |  |  |  | + | - | - |  |
| En | Mod59 | 3rd | 3R | 92A | *Dl* | + | - | - |  |
| En | Mod61 | 2nd |  |  |  | + | - | - |  |
| En | Mod76 | 2nd |  |  |  | + | - | - |  |
| En | Mod90 | 3rd | 3L | 66D | *msk* | - | + | ++++ |  |
| En | Mod111 | 3rd | 3R | 92A | *Dl* | + | - | - |  |
| En | Mod121 | 2nd |  |  |  | + | - | - |  |
| Su+ | Mod130 | 3rd | 3R | 92A | *Dl* | + | - | - |  |
| Su+ | Mod140 | 3rd | 3R | 92A | *Dl* | + | - | - |  |
| ***Dys^E6^*** | | | | | | | | | |
| En | ModE10 | 3rd |  |  | *Dys* | + | - | - |  |
| Mod | ModE11 | 2nd |  |  |  | - | + | ++++ | 60+ days |
| Mod | ModE21 | 3rd |  |  |  | + | - | - |  |
| Mod | ModE26 | 3rd |  |  |  | + | - | - |  |

| a. phenotypic groups are indicated in Figure 3. |
| --- |
| b. E denotes modifier of the E6 deletion of dystrophin. Since E6 gives wild type wing veins as heterozygotes, these modifiers are enhancers. Su denotes |
| suppressor. |
| c. homozygous lethal indicates that homozygotes cannot be constructed. () denote semi-lethality. |
| d. homozygous fertile indicates that homozygotes can be maintained as a stock |
| e. dominant wing vein phenotype indicates that the lesion produces a phenotype in the absence of a dystrophin mutant ++++ indicates 100% penetrance |
| () denote less than 25% penetrance. |
| f. recessive wing vein phenotype indicates that the lesion produces a phenotype in the homozygous state ++++ indicates 100% penetrance. |
| () denotes the phenotypic group of the homozygotes |
| g. lifespan for wild type flies is around 70 days at room temperature |
