# Supplementary figures and images for: Genetic Modifier Screens Reveal New Components that Interact with the Drosophila Dystroglycan-Dystrophin Complex
Source: PLoS One. 2008 Jun 11;3(6):e2418. doi: 10.1371/journal.pone.0002418 (PMC2398783; doi:10.1371/journal.pone.0002418)

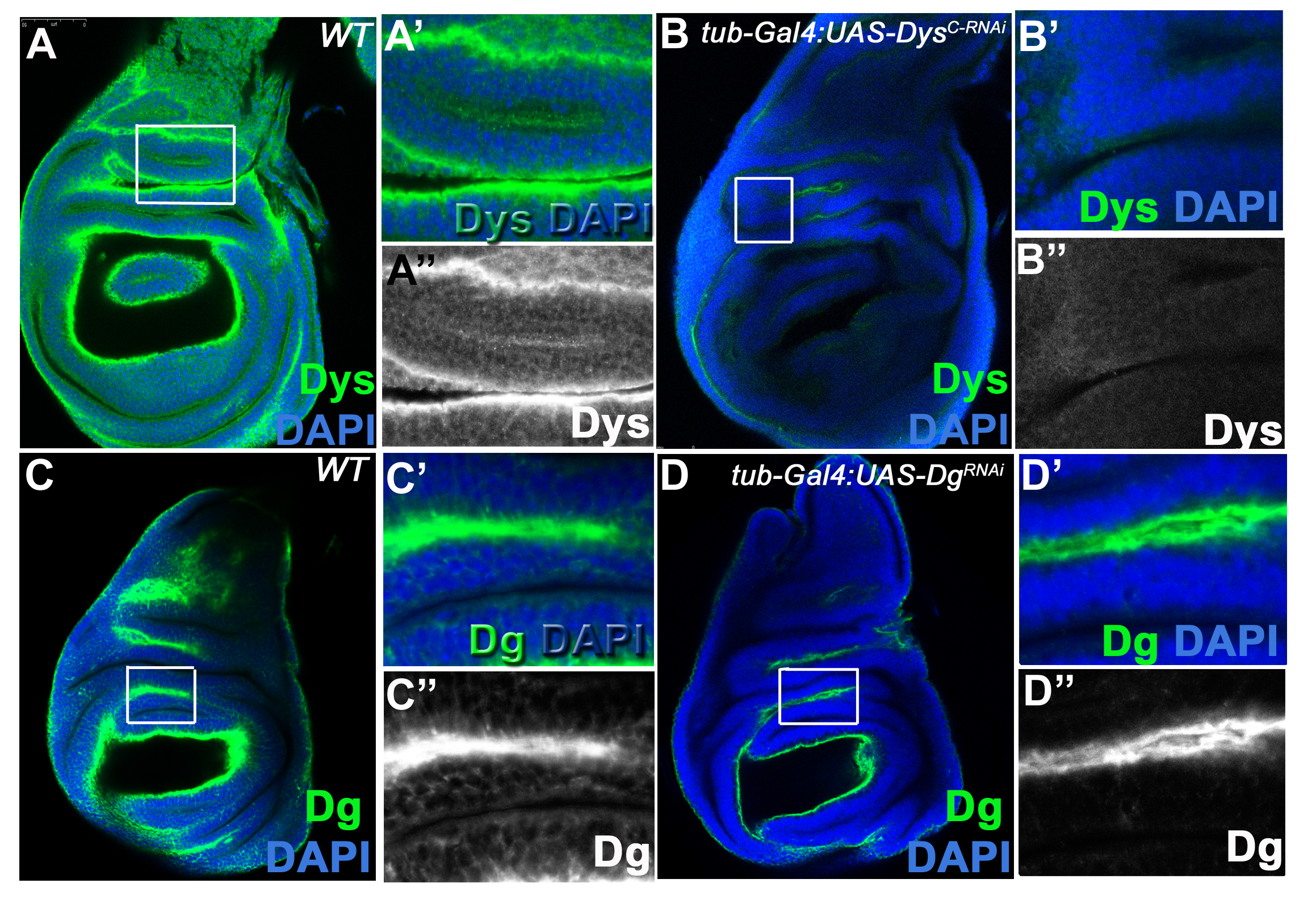

Supplement: Figure S1 — Dys and Dg Expression in Wild Type and Mutant Wing Discs. In wild-type larvae, Dys is expressed in all cells of the wing disc (A) and is strongly reduced in the wing disc of the DysC-RNAi mutant (B; tubGal4:UAS-DysC-RNAi/+). Dystroglycan localization in wild type imaginal discs is enriched at the basal surface of the epithelial cells. Dg expression is more intense in folds formed from the contact of basal surfaces and is less visible in the folds made from apical surfaces of wing disc cells (C). The DgRNAi mutant also shows a reduction of Dystroglycan protein in the wing disc (D; tub-Gal4:UAS-DgRNAi/+). (A′–D′) Enlarged images of framed areas on (A–D). (A"–B") Enlarged images of framed areas on (A–B) show Dystrophin single channel staining. (C"–D") Enlarged images of framed areas on (C–D) show Dystroglycan single channel staining. (6.06 MB TIF) [file pone.0002418.s003.tif]

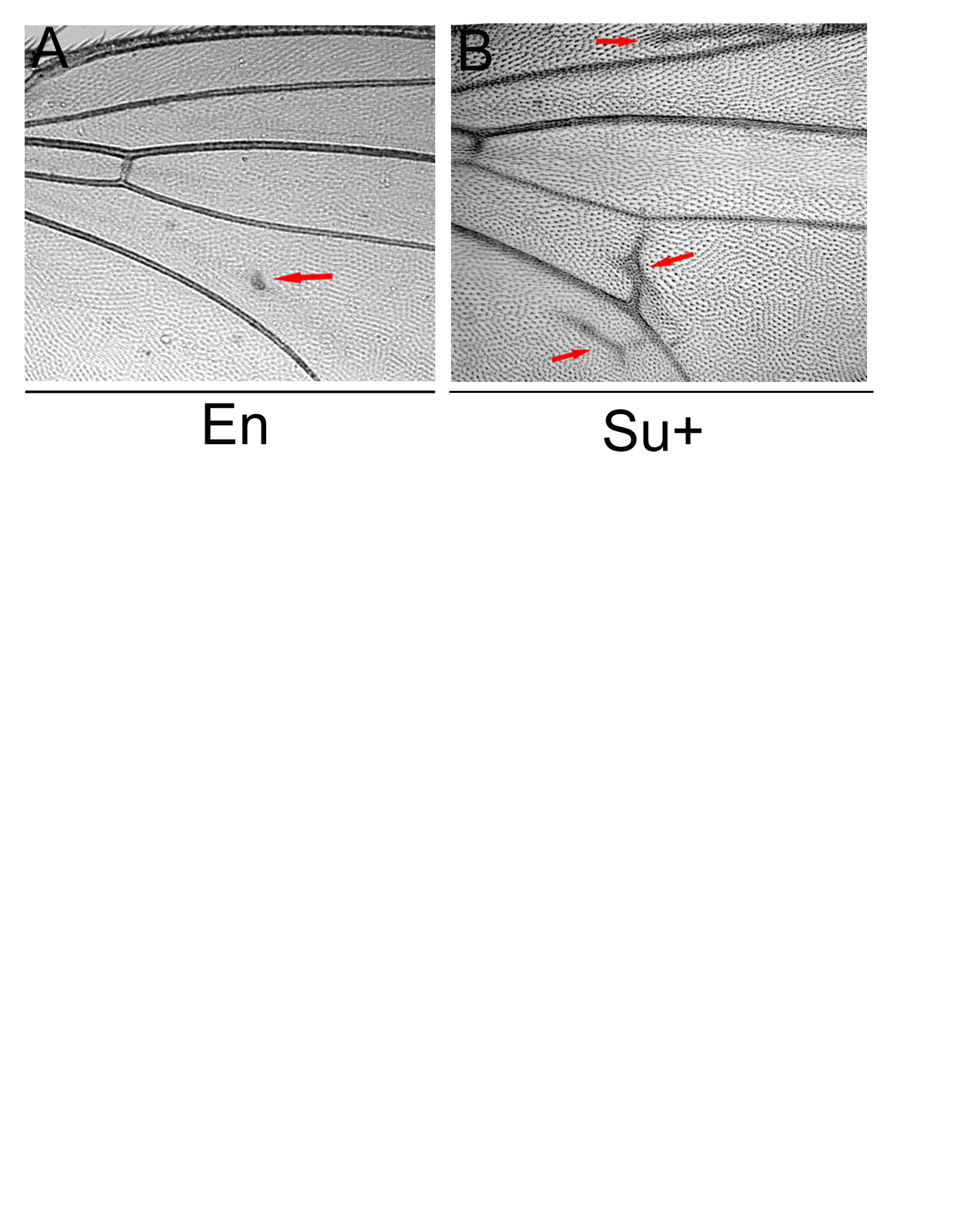

Supplement: Figure S2 — Subclasses of Dys(RNAi) Modifiers. (A) Example of enhancers that lack the posterior cross vein (PCV) and belong to phenotypic class En. Arrow indicates where the PCV should be. (B) Wing representing Su+ class phenotype that shows the PCV attached to L4 and L5 and extra wing vein material can be seen below L5. (0.94 MB TIF) [file pone.0002418.s004.tif]

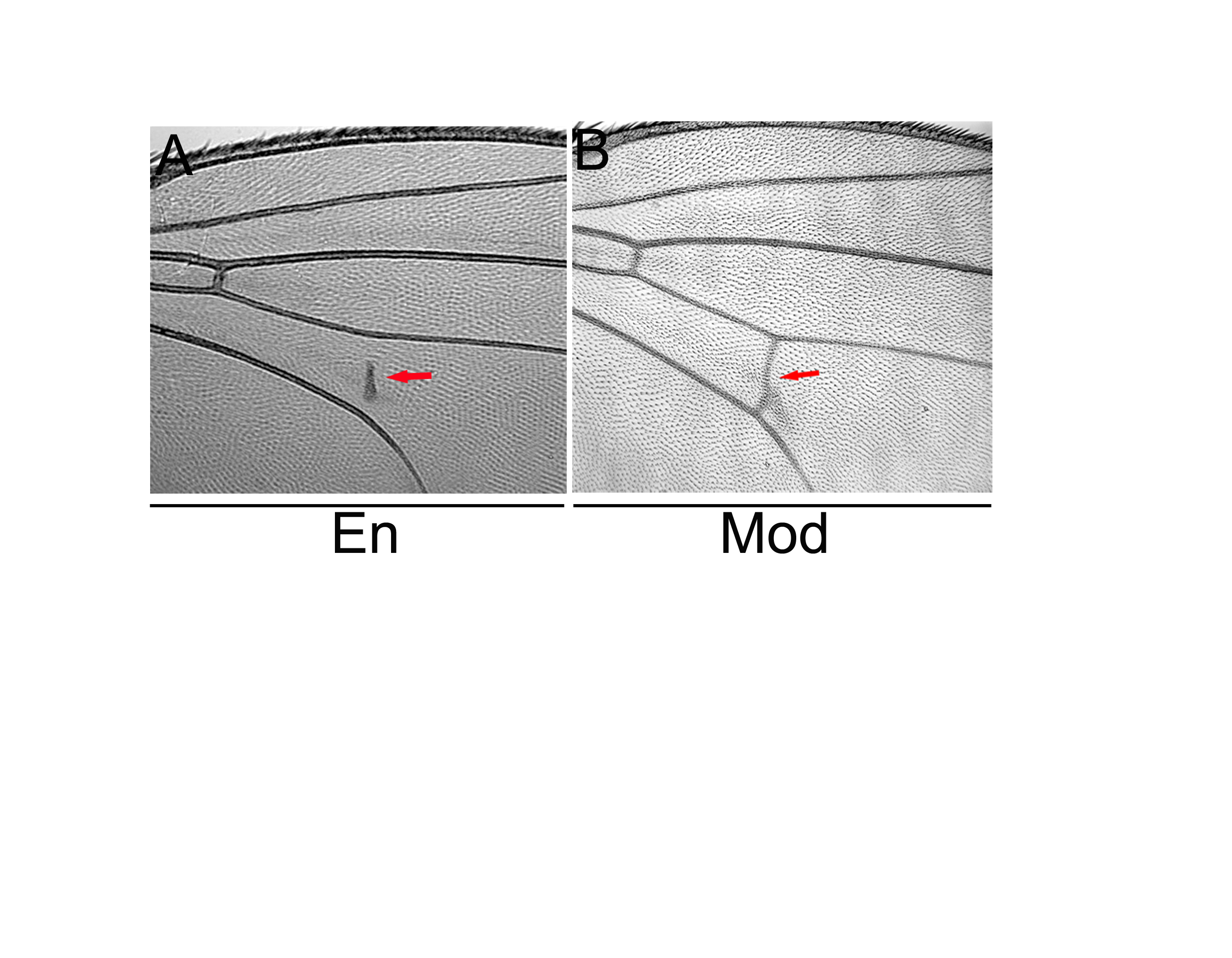

Supplement: Figure S3 — DysE6/+ Modifiers. DysE6/+ flies have normal posterior cross veins. (A) Represents an enhancer (ModE10) of this phenotype which phenocopies the wing veins from DysE6/DysE6 flies. (B) Shows an extra vein modification of the DysE6/+ posterior cross vein. Arrows indicate altered cross veins. (1.94 MB TIF) [file pone.0002418.s005.tif]

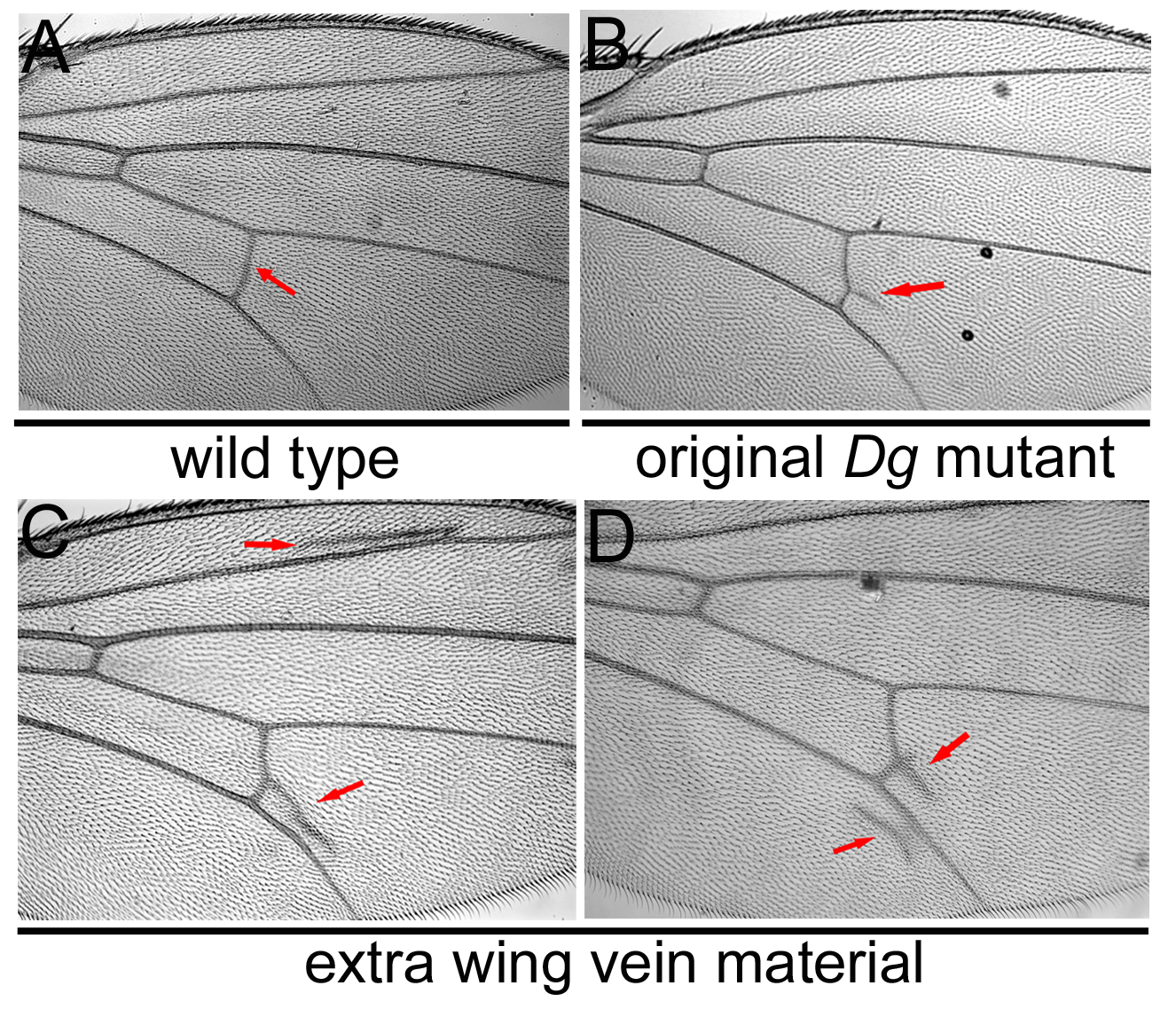

Supplement: Figure S4 — Dg(RNAi) Modifiers. (A) Wild type fly wing with normal posterior cross vein (PCV) as indicated by the arrow. (B) DgRNAi mutant PCV (arrow) with a branch. (C–D) represent modifiers that produce extra vein material (indicated by arrows. In one case (C) the branch is elongated with extra material also seen above L2 (upper arrow). (D) Shows extra material below L5 (lower arrow). (1.77 MB TIF) [file pone.0002418.s006.tif]

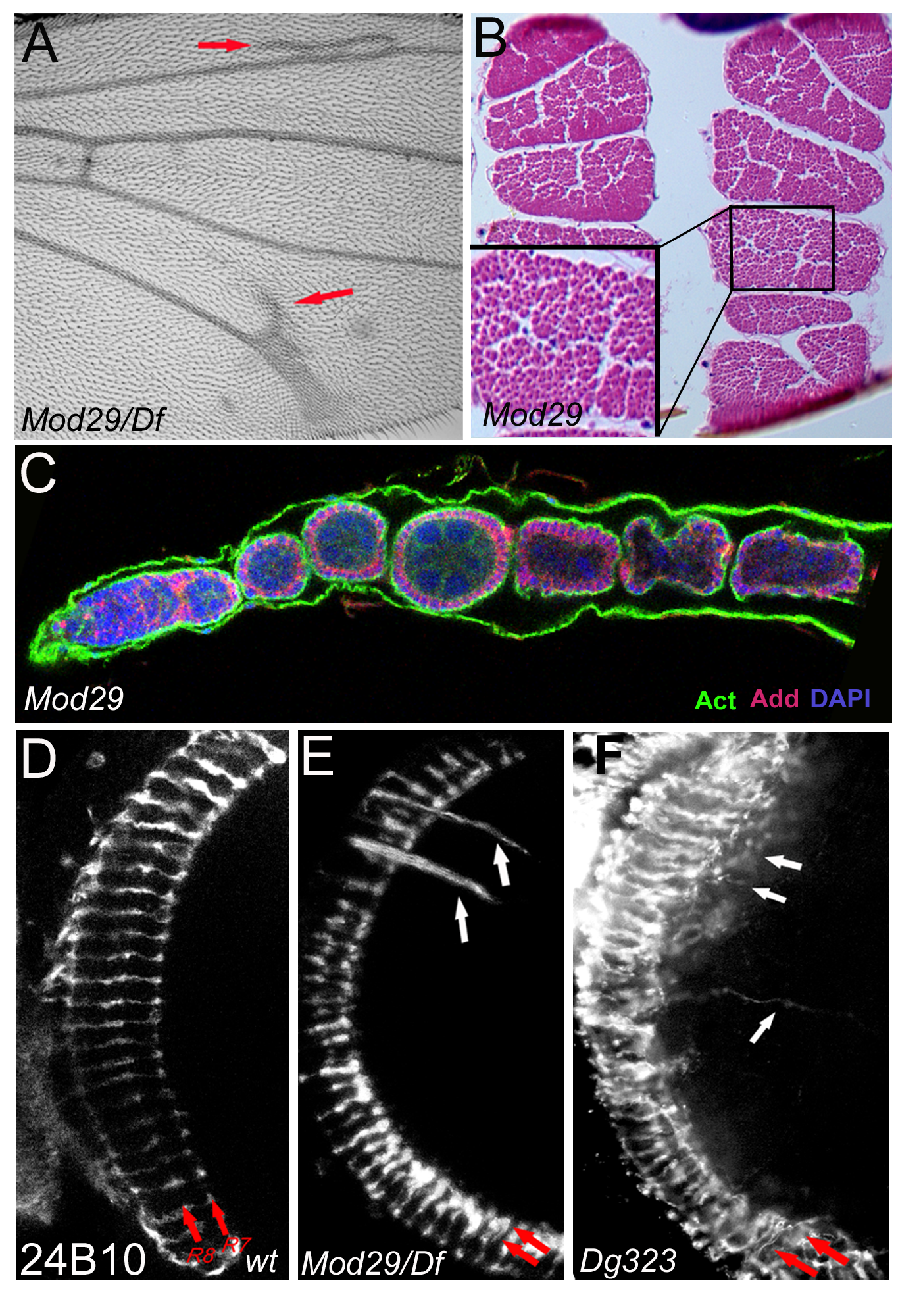

Supplement: Figure S5 — Phenotypes Observed in poly-EGF Mutants. (A) Fly wing from Mod29/Df flies. Arrows indicate extra vein material. (B) Transverse histological section of the indirect flight muscle in Mod29 homozygotes. (B') Higher magnification of the region indicated by the box which indicates the separation of the muscle into individual fibers. (C) Ovariole from a Mod29 homozygote. Anterior to the left. The germarium is at the anterior most tip of the ovariole with developing egg chambers progressing to the right (posteriorly). The egg chambers undergo developmental arrest in later stages. Actin-Green, Adducin-Red, DAPI-purple. (D) 24B10 antibody staining of the adult wild type brain. Arrows indicate photoreceptor axon termination sites where the R8 photoreceptor axon (left arrow) terminates before the R7 axon (right arrow). (E) Mod29/Df adult brain. Termination of the R8 and R7 axons are indicated by the red arrows. White arrows indicate non termination of two axons that protrude deeper into the brain. (F) Dg323/Dg323 clone in the adult brain. Red arrows indicate where the R8 and R7 axons should terminate. Upper white arrows indicate a general disruption of axon termination in the R8/R7 termination region. Lower right arrow indicates a non terminating axon that proceeds deeper into the brain. (3.58 MB TIF) [file pone.0002418.s007.tif]
